# Heteroresistance associated with the production of fosfomycin-resistant inner colonies during disk diffusion testing among a geographically diverse collection of *Klebsiella pneumoniae* clinical isolates

**DOI:** 10.1093/jacamr/dlaf013

**Published:** 2025-02-20

**Authors:** Morgan L Bixby, Lindsey B Collins, Ellora C Daley, Jenna M Salay, Sofia Oliver, Alexandra L Bryson, Elizabeth B Hirsch

**Affiliations:** Department of Experimental and Clinical Pharmacology, University of Minnesota College of Pharmacy, Minneapolis, MN, USA; Department of Experimental and Clinical Pharmacology, University of Minnesota College of Pharmacy, Minneapolis, MN, USA; Department of Experimental and Clinical Pharmacology, University of Minnesota College of Pharmacy, Minneapolis, MN, USA; Department of Experimental and Clinical Pharmacology, University of Minnesota College of Pharmacy, Minneapolis, MN, USA; Department of Experimental and Clinical Pharmacology, University of Minnesota College of Pharmacy, Minneapolis, MN, USA; Department of Pathology, Virginia Commonwealth University Health System, Richmond, VA, USA; Department of Experimental and Clinical Pharmacology, University of Minnesota College of Pharmacy, Minneapolis, MN, USA

## Abstract

**Background:**

Fosfomycin susceptibility breakpoints apply only to *Escherichia coli* despite clinical use against *Klebsiella pneumoniae.* EUCAST and CLSI have different breakpoints and guidelines for disk diffusion (DD) interpretation that are frequently extrapolated to *K. pneumoniae.* Guidelines differ in interpreting inner colonies (IC) that grow within the zone of inhibition, but specificity to *E. coli* leaves knowledge gaps when extrapolating to other uropathogens.

**Objectives:**

To examine the frequency and MIC of *K. pneumoniae* IC during fosfomycin DD testing and to determine potential relationships between IC production, heteroresistance and *fosA* presence.

**Methods:**

A collection of *K. pneumoniae* clinical isolates (*n* = 262) and their IC (*n* = 116) underwent broth microdilution testing. Heteroresistance screening and PCR for *fosA* was performed on susceptible isolates that either never produced (NP) IC (*n* = 14) or produced ≥5 resistant IC (*n* = 43).

**Results:**

The MIC range (≤2 to >256 mg/L) of clinical isolates increased to 32 to >1024 mg/L for the IC collection with a median MIC increase of three, 2-fold dilutions. IC producers had 1.71 greater odds (*P* < 0.01) of a positive heteroresistance screen compared to NP isolates. No relationship was found between *fosA* presence and either IC production or heteroresistance.

**Conclusions:**

Production of ≥5 IC among clinical *K. pneumoniae* isolates was frequent and often resulted in an increased IC isolate MIC. Significantly greater odds of heteroresistance among IC producers were found when compared to NP isolates. Thus, presence of IC during fosfomycin DD testing should prompt avoidance of fosfomycin treatment.

## Introduction

Fosfomycin susceptibility testing recommendations are complex. Although agar dilution (AD) is considered the reference method by both Clinical and Laboratory Standards Institute (CLSI) and the European Committee on Antimicrobial Susceptibility Testing (EUCAST), it is time- and resource-intensive.^1,2^ Alternative options including broth microdilution (BMD) and disk diffusion (DD) are often used in clinical microbiology laboratories.^3–20^ While DD is an approved method for *Escherichia coli* testing from both CLSI and EUCAST, a modified BMD protocol has received FDA clearance as an addition to the Vitek 2 (bioMérieux) Gram negative card in the USA.^1,2,21–23^ All three methods pose problems; specifically, AD and BMD are prone to similar rates of scientific error (i.e. variability) that result in higher minimal inhibitory concentration (MIC) from BMD.^24–27^Fosfomycin DD often produces inner colonies (IC) of unknown origin, of which CLSI and EUCAST protocols have contradictory recommendations for interpretation of *E. coli* isolates.^27–30^ CLSI states that the zone of inhibition shall be measured from the innermost colonies, whereas EUCAST states that the inner colonies shall be ignored.^1,31^ These IC have been noted to occur at varying rates for different organisms among different studies ranging from 3.1% to 69% in *E. coli* and 19% to 82.5% in *K. pneumoniae.*^27,28,30^ The occurrence of IC in susceptibility testing suggest these clinical samples are a heteroresistant culture. Heteroresistance is defined as pre-existing subpopulation of resistant cells caused by either varying expression of or stable mutations within either resistance genes or genes that influence changes in antibiotic resistance.^32^

In 2018, a CLSI *ad hoc* working group (AHWG) was convened to evaluate the issue of IC during fosfomycin testing. After literature review—which focused on one paper^28^ available at that time—the AHWG stated that IC were rare among the *E. coli* isolates for which these breakpoints apply (https://www.idsociety.org/idsa-newsletter/september-26-2018/clsi-updates-from-the-clsi-subcommittee-on-susceptibility-testing). Both CLSI and EUCAST state that fosfomycin breakpoints should not be extrapolated to non-*E. coli* Enterobacterales. However, in real-world settings, these breakpoints are extrapolated for use against other non*-E. coli* organisms that left the AHWG concerned that methods ignoring the IC would be extrapolated to organisms with more prevalent rates of IC production and additional resistance genes (e.g. *fosA*).^24–26,28–30^ Among these clinical studies where fosfomycin monotherapy was used to treat *K. pneumoniae* urinary tract infections (UTI), ∼50% or more of the patients experienced some type of treatment failure despite the fact that isolates were susceptible per the extrapolated *E. coli* breakpoints.^33–37^ Previous investigations have implicated *fosA* genes as a major source of fosfomycin resistance among *K. pneumoniae* due to the presence of both chromosomal and plasmid-mediated variations in the *K. pneumoniae* population.^29,38–44^

To understand contributors to the discordance between *in vitro* susceptibility testing results and *in vivo* clinical outcomes for fosfomycin treatment of *K. pneumoniae*, we sought to determine the frequency and MIC values of IC compared to their IC-producer cultures among a geographically diverse collection of isolates (*n* = 262). We also investigated whether the heteroresistance status and presence of *fosA* among the clinical isolate collection contributed to the production of IC.

## Methods

### Bacterial isolates

This study included 262 *K. pneumoniae* clinical isolates collected from five US locations during 2013–2016 (*n* = 80)^26,27,45–47^ and 2022–2023 (*n* = 182), henceforth referred to as the ‘clinical isolate collection’, as well as the IC produced by these isolates during previous disk diffusion testing in our research laboratory (*n* = 116).^26,27^ The presence of IC for the purposes of this study was defined as ≥5 IC present within at least one single DD replicate. As colony morphology was similar for all IC, the closest IC to the disk was selected for subsequent subculturing, testing and long-term storage at −80°C. Anatomical culture sites of the isolates included urine (*n* = 245), blood (*n* = 9), sputum (*n* = 5) and wounds (*n* = 3). Of these, 48 (18.3%) were confirmed to be ESBL-producing isolates by the hospital microbiology laboratories of origin.^26,27,45–47^ The presence of these IC subpopulations created three distinct phenotypes including: isolates that never produced an IC (‘never producer’ or NP), isolates that had IC arise during DD testing (IC producer) and the visually distinct IC themselves.

### Susceptibility testing and interpretation

Fosfomycin susceptibility was determined in biological duplicate, on separate days, via both AD and BMD for the clinical isolate collection and only BMD for the IC collection.^1,26,27^ BMD was chosen as the MIC comparison method due to the resource-intensive limitations of AD, especially for isolates with elevated MIC. For both AD and BMD, isolates were tested on individual days in technical triplicate. The MIC for each test was read as the first concentration where there was no visible growth for all three technical replicates. The final MIC used for analysis was the modal MIC accounting for acceptable error of ±1 dilution.

Test isolates (clinical isolate collection: *n* = 262; IC collection: *n* = 116) were inoculated onto blood agar plates for overnight growth at 37°C. Single isolated colonies were inoculated into sterile saline to obtain a density of ∼1.5 × 10^8^ cfu/mL. For AD, the saline suspension was diluted to 2 × 10^6^ cfu in Mueller–Hinton II broth (MHB) (Fisher Scientific; Hampton, NH, USA) where 5 µL (10^4^ cfu) of the MHB suspension was placed in triplicate onto a series of Mueller–Hinton agar (Fisher Scientific; Hampton, NH, USA) plates supplemented with fosfomycin (Fisher Scientific; Hampton, NH, USA) plus 25 mg/L of glucose-6-phosphate (G6P) (Neta Scientific Inc.; Hainesport, NJ, USA). For BMD, the saline suspension was diluted to 10^6^ cfu/mL in MHB where 50 µL of the MHB suspension was placed into three columns of a 96-well plate with each row containing a serial dilution of fosfomycin plus 50 mg/L (final concentration 25 mg/L) of G6P. Test concentrations of fosfomycin ranged from 2 to 256 mg/L for the clinical isolate collection and 8 to 1024 mg/L for the IC isolate collection.^1,27^  *Enterococcus faecalis* ATCC 29212 (MIC: 32–128 mg/L) was used as a control strain, per Table 5A-1 of the CLSI M100 to capture the typical concentration range of our test isolates and was run in parallel with test isolates for each susceptibility test. Furthermore, to ensure appropriate G6P concentrations, *E. coli* ATCC 25922 was used to test each new batch of frozen G6P with fosfomycin using AD to confirm MIC with G6P potentiation.

Owing to the lack of established fosfomycin breakpoints for *K. pneumoniae*, CLSI and EUCAST *E. coli* breakpoints were extrapolated (Table 1). The EUCAST *MIC Distributions for Fosfomycin* database identifies the epidemiological cut-off value (ECOFF) for fosfomycin against *K. pneumoniae* at 128 mg/L, which was used to assess the wild-type distribution among our isolate collection in addition to extrapolated susceptibility.^48^

**Table 1.** CLSI and EUCAST breakpoints for oral and intravenous (IV) fosfomycin formulations

	MIC (mg/L)		
	Susceptible	Intermediate	Resistant
CLSI^a^	≤64	128	≥256
EUCAST^b^	≤8	NA	>8

^a^CLSI *M100, Thirty-Fourth Edition* applies only to *E. coli* from the urinary tract and *E. faecalis.*

^b^EUCAST oral breakpoints apply only to uncomplicated UTI caused by *E. coli*; intravenous breakpoints only *E. coli* infections originating in the urinary tract.

### Heteroresistance screening

A modified disk elution screening test from Abbott *et al.* was performed on a subset of the IC producers and IC isolates to compare the frequency of heteroresistance between the susceptible isolates (per extrapolated CLSI breakpoints) that produced ≥5 IC (*n* = 43) and NP (*n* = 14).^24^ Per the recommendation of Abbott *et al*. (via personal communication), the disk elution test was conducted using six commercially available fosfomycin disks (200 µg fosfomycin, 50 µg glucose-6-phosphate) (Becton and Dickinson, Franklin Lakes, NJ, USA), an increase from their initial study using two or four disks, which were added to 1.9 mL of MHB in a test tube and allowed to elute for 90 minutes to approximate a fosfomycin concentration of ∼420 mg/L (68–70% of the total disk fosfomycin volume; as quantified by Abbott *et al.*).^24^ Overnight broth cultures containing ∼10^8^ cfu/mL were used to inoculate both a disk-free control and the experimental tubes at 100 µL. Tubes were incubated at 37^○^C for 72 hours and assessed for turbidity visible to the unaided eye. A tube that was turbid to the unaided eye was considered to be an isolate that screened positive for heteroresistance. An internal IC strain (B128D) with an MIC >1024 mg/L was used as a positive growth control, *E. coli* ATCC 25922 (MIC 0.5–2 mg/L) as a negative growth control and a bacteria-free negative control were run in parallel with test isolates.

**Figure 1.**
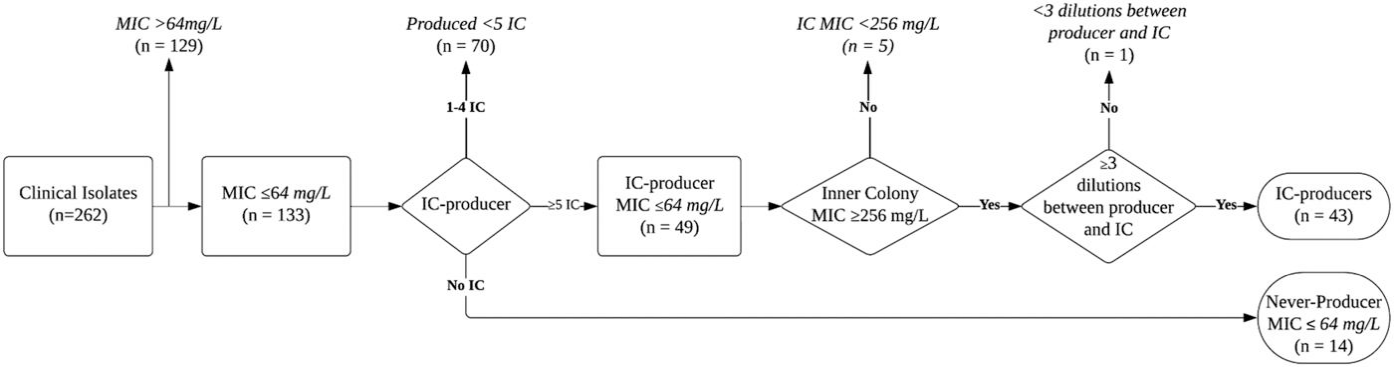
Flowchart of inclusion criteria for the heteroresistance screening. All MIC data are based on broth microdilution results.

Isolates were included in the screening if they were susceptible per extrapolated CLSI breakpoints (≤64 mg/L) and either an IC producer or NP. IC producers needed to meet additional criteria of having a resistant IC (per extrapolated CLSI breakpoints,  ≥ 256 mg/L), producing ≥5 IC, and having a difference in MIC of at least three 2-fold dilutions between the IC producer and its IC (Figure 1).

Results underwent linear regression to determine the odds ratio for heteroresistance and being an IC producer. Statistical data were processed using R version 4.3.2 and Rstudio Desktop version 2023.12.1 (Rstudio, Boston, MA, USA). Packages used were the stats version 4.3.2 and base version 4.3.2.

### *Presence of* fosA

Isolates that met the same inclusion criteria as the heteroresistance screening were tested for the presence of *fosA* genes. DNA extractions were performed using the DNeasy mini kit (QIAGEN; Hilden, Germany). The presence of *fosA* was determined via PCR amplification using primers for *fosA* (forward GAGCGTGGCGTTTTATCAGC; reverse GCCTCGCACTACTTCCTCGA) and *fosA3* (forward GCGTCAAGCCTGGCATTT; reverse GCCGTCAGGGCTGAGAAA.^13^ A *fosA* containing *K. quasipneumoniae* (ATCC 700603) was uploaded to Geneious Prime (Dotmatics; Boston, MA, USA) to create sequence-specific *fosA* primers. The location of *fosA* primers was confirmed using a reference sequence (NZ_CP073236.1) that is isolated from a *Klebsiella* species. Primers were created by highlighting the gene sequence and using the ‘Design new primers’ tool. Both sets of primers were tested against the *K. pneumoniae* (ATCC 13883) genome sequence for specificity. The amplification product was run on gel electrophoresis for presence of an amplicon of ∼267 and 282 bp for *fosA* and *fosA3*, respectively.^13^) To confirm the amplified sequences were correct, PCR product of three isolates that had electrophoresis bands of the target size of each gene were sent to ACGT DNA Sequencing Services (Wheeling, Illinois, USA) for Sanger sequencing. The Sanger sequence results from both forward and reverse primers were then mapped to the reference genome (ATCC 700603) to ensure that the sequences aligned with the target gene. The presence of *fosA* and *fosA3* genes were also analysed in relation to positive heteroresistance screenings via Fisher’s exact test.

## Results

### Susceptibility testing and interpretation

The BMD MIC values ranged from ≤2 to >256 mg/L for the clinical isolate collection (*n* = 262) and 32 to >1024 mg/L for IC (*n* = 116) (Table 2). By extrapolated *E. coli* breakpoints, 50.8% (*n* = 133) of the clinical isolate collection were susceptible by CLSI breakpoints as well as 3.8% (*n* = 10) by EUCAST breakpoints. The EUCAST ECOFF for fosfomycin against *K. pneumoniae* placed 71.0% (*n* = 186) of the collection as wild-type isolates. The MIC_50/90_ for this collection was 64/256 mg/L.

**Table 2.** Interpretation of MIC fosfomycin susceptibility testing results using extrapolated *E. coli* breakpoints for clinical isolates and IC

	Susceptible *n* (%)	Intermediate *n* (%)	Resistant *n* (%)	MIC range (mg/L)	MIC_50/90_ (mg/L)
Clinical isolate collection (*n* = 262) using agar dilution					
CLSI	203 (77.5)	21 (8.0)	38 (14.5)	1 to >256	32/256
EUCAST	22 (8.4)	NA	240 (91.6)		
Clinical isolate collection (*n* = 262) using broth microdilution					
CLSI	133 (50.8)	53 (20.2)	76 (29.0)	≤2 to >256	64/256
EUCAST	10 (3.8)	NA	252 (96.2)		
Inner colony isolates (*n* = 116) using broth microdilution					
CLSI	4 (3.4)	3 (2.6)	109 (93.9)	32 to >1024	>1024/>1024
EUCAST	0 (0.0)	NA	116 (100.0)		

**Figure 2.**
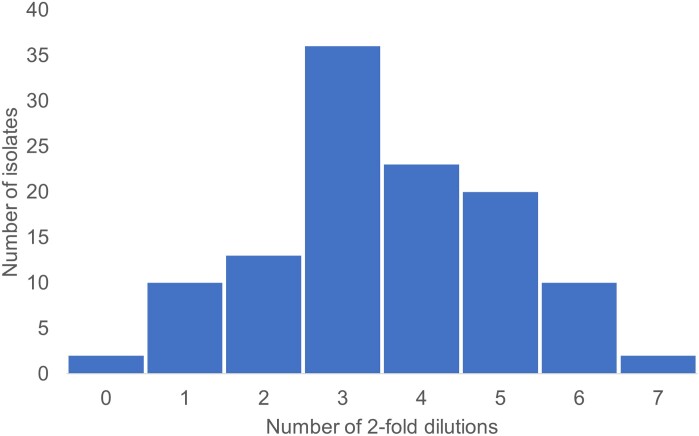
Number of 2-fold dilution increase in MIC value of inner colony isolates arising from each clinical isolate.

The IC collection had 93.9% (*n* = 109) resistant isolates per CLSI and 100% (*n* = 116) per EUCAST extrapolated *E. coli* breakpoints. Using the EUCAST ECOFF, only 6.1% (*n* = 7) of IC isolates were identified as wild type. The MIC_50/90_ for this collection was >1024/>1024. IC isolates had an MIC that was up to seven, 2-fold dilutions higher than their respective IC producers and a modal increase of three dilutions (Figure 2). An increase in MIC of two or more 2-fold dilutions was seen in 89.7% of isolates as two isolates had no increase in MIC and another 10 isolates had an increase of a single 2-fold dilution, which is an acceptable error.

### Heteroresistance screening

Of the susceptible collection of IC-producer strains, 95.3% (*n*/*N *= 41/43) screened positive for heteroresistance (Table 3). The two isolates that had a negative screening were M063 [MIC (IC producer (P)/IC): 32/1024 mg/L] and V057 [MIC (P/IC): 64/>1024 mg/L]. Among the NP isolates, 64.3% (*n*/*N *= 9/14) screened positive for heteroresistance. The NP isolates that screened negative had an MIC of 8 mg/L (B254, M041, M073), 32 mg/L (M048) and 64 mg/L (M055). The difference in rates of a positive heteroresistance screening was significant (*P* < 0.01). Additionally, IC producers had 1.71 greater odds (95% CI 1.24−2.34; *P* < 0.01) of screening heteroresistant compared to NP isolates.

**Table 3.** Heteroresistance screening and the amplification of fosA for the clinical (*n* = 57) isolate collection, which was composed of the IC producers (*n* = 43) and never-producers (*n* = 14), along with the IC (*n* = 43) isolate collections

	IC-producer status	Broth microdilution MIC (mg/L)		Heteroresistance screening	*fosA*		*fosA*3	
		clinical	IC		Clinical	IC	Clinical	IC
V044	Y	8	512	+	+	+	−	−
B131	Y	8	1024	+	+	+	−	−
B035	Y	16	1024	+	+	+	−	−
V046	Y	16	1024	+	−	−	−	−
M009	Y	16	>1024	+	+	+	−	−
B069	Y	32	256	+	+	+	−	−
M049	Y	32	256	+	+	+	−	−
B068	Y	32	1024	+	+	+	−	−
M063	Y	32	1024	−	+	+	−	−
V025	Y	32	1024	+	+	+	−	−
B120	Y	32	>1024	+	+	+	−	−
M001	Y	32	>1024	+	+	+	−	−
V001	Y	32	>1024	+	−	−	−	−
V033	Y	32	>1024	+	+	+	−	−
V094	Y	32	>1024	+	+	+	−	−
B019	Y	64	512	+	−	+	−	−
B090	Y	64	512	+	+	+	−	−
M021	Y	64	512	+	+	+	−	−
V065	Y	64	512	+	+	+	−	−
V083	Y	64	512	+	+	+	−	−
B093	Y	64	1024	+	+	+	−	−
B096	Y	64	1024	+	+	+	−	−
B121	Y	64	1024	+	+	+	−	−
B137	Y	64	1024	+	+	+	−	−
P550	Y	64	1024	+	+	+	−	−
V085	Y	64	1024	+	+	−	−	−
B132	Y	64	>1024	+	+	+	−	−
B209	Y	64	>1024	+	+	+	−	−
B233	Y	64	>1024	+	+	+	−	−
M054	Y	64	>1024	+	+	+	−	−
M058	Y	64	>1024	+	+	+	−	−
P570	Y	64	>1024	+	+	+	−	−
P615	Y	64	>1024	+	+	+	−	−
V008	Y	64	>1024	+	+	+	−	−
V014	Y	64	>1024	+	+	+	−	−
V018	Y	64	>1024	+	+	+	−	−
V027	Y	64	>1024	+	+	+	-	-
V037	Y	64	>1024	+	+	+	−	−
V055	Y	64	>1024	+	+	+	−	−
V057	Y	64	>1024	−	+	+	−	−
V058	Y	64	>1024	+	+	+	−	−
V076	Y	64	>1024	+	+	+	−	−
V096	Y	64	>1024	+	+	+	−	−
B254	N	8	NA	−	−	NA	−	NA
B275	N	8	NA	+	+	NA	−	NA
M041	N	8	NA	−	+	NA	−	NA
M073	N	8	NA	−	+	NA	−	NA
V078	N	16	NA	+	+	NA	−	NA
M048	N	32	NA	−	+	NA	−	NA
P653	N	32	NA	+	+	NA	−	NA
M010	N	64	NA	+	+	NA	−	NA
M011	N	64	NA	+	+	NA	−	NA
M055	N	64	NA	−	+	NA	−	NA
P628	N	64	NA	+	+	NA	−	NA
P531	N	64	NA	+	+	NA	−	NA
P547	N	64	NA	+	+	NA	−	NA
V016	N	64	NA	+	+	NA	−	NA

N, no; NA, not applicable; Y, yes.

### *Presence of* fosA*/*fosA3 *genes*

Of the NP isolates, 92.9% (*n*/*N* = 13/14) were *fosA* positive per PCR amplification with the primer set used. Isolate B254 was the only NP isolate that screened negative for heteroresistance and was also negative for *fosA* amplification. There was no significant relationship between heteroresistance and *fosA* in NP (*P* = 0.36). None of the NP isolates contained a *fosA3* plasmid per PCR amplification with the primer set used.

Of the IC producers, 93.0% (*n*/*N* = 40/43) were *fosA* positive per PCR amplification. None of the isolates that were negative for *fosA* amplification were also negative for heteroresistance screening. There was no significant relationship between heteroresistance and *fosA* in IC producers (*P* = 1). None of the IC producers contained a *fosA3* plasmid per PCR amplification.

Of the IC isolates, 93.0% (*n*/*N* = 40/43) were *fosA* positive per PCR amplification. Two of the *fosA* negative IC (V001D and V046D) arose from IC producers that were also negative for *fosA* amplification. Isolate V085D was negative for *fosA* amplification, but the corresponding IC producer was positive for *fosA* amplification. Another isolate (B019D) was positive for *fosA* amplification, but the corresponding IC producer was negative for *fosA* amplification. There was no significant relationship between the presence of *fosA* in the IC and heteroresistance of the IC producers (*P* = 1). None of the IC isolates contained a *fosA3* plasmid per PCR amplification.

## Discussion

In addition to the occurrence of more frequent IC, the presence of inherent *fosA* genotypes, and noted heteroresistance are cited reasons why oral fosfomycin may be considered a poor treatment option for *K. pneumoniae* UTI.^24,29^ This study sought to compare the frequency and MIC of the IC arising from fosfomycin susceptibility testing as compared to their IC producers. This was further expanded to explore the rates of heteroresistance among IC producers and NP, as well as the presence of *fosA* among all three phenotypes (NP, IC producers and IC).

While the interpretation of the susceptibility testing differs by the breakpoints extrapolated from *E. coli*, the MIC range of the clinical isolate collection (*n* = 262) was lower (≤2 to >256 mg/L) as compared to the IC collection (*n* = 116, 32 to >1024 mg/L). The modal increase of three 2-fold dilutions from IC producer to IC was also reflected in the MIC_50/90_ of 64/256 mg/L and >1024/>1024 mg/L for the clinical isolate and IC collections, respectively. Furthermore, most of the IC collection (93.9%, *n*/*N *= 109/116) was not part of a wild-type population differing from most of the clinical isolate collection that were wild type (71.0%, *n*/*N* = 183/262) per the EUCAST ECOFF.

Despite different IC phenotypes, both the IC producers and NP had isolates that screened positive for heteroresistance using the modified screening test. IC producers had greater odds (1.71; 95% CI 1.24−2.34; *P* < 0.01) of having a positive heteroresistance screening as compared to the NP. Despite the significant difference in heteroresistance between IC producers and NP, there was still a large proportion of NP (64.3%) that also screened positive for heteroresistance. It is possible that these isolates had expression of a fosfomycin resistance gene expressed in the much higher fosfomycin concentration as compared to typical DD, whereas the IC producers already contain a subpopulation (IC) that are more resistant to fosfomycin due to either varying expression of fosfomycin resistance genes or variations in the genome itself. Heteroresistance appeared to be unrelated to *fosA* among the clinical isolate collection, as there was no significant relationship between heteroresistance and presence of *fosA* in IC producers (*P* = 1) or NP (*P* = 0.36). Additionally, a single IC that arose from *fosA* positive IC producers (*n* = 40) lost or did not have an intact chromosomal *fosA* gene. The *fosA3* gene, which is carried on plasmids, was not present in any of the isolates.

Mojica *et al.* conducted BMD and DD testing on an isolate collection including *K. pneumoniae* isolates (*n* = 68).^30^ Of their collection, 76% and 85% of isolates were susceptible per EUCAST and CLSI breakpoints, respectively. The authors reported lower MIC_50/90_ values (8/32 mg/L) compared to the current study (64/256 mg/L). In addition to the lower MIC, the authors noted a lower incidence of IC (19%) as compared to the current study (44.3%). Elliott *et al.* also noted that IC were ‘frequently present’ for non-*E. coli* species during fosfomycin DD testing among a collection of *Klebsiella pneumoniae* carbapenemase (KPC)-producing isolates.^29^ All KPC-producing isolates (*n* = 24) in their collection carried intrinsic *fosA* per whole-genome sequencing, fairly similar to the 93.0% (*n*/*N* = 53/57) within the clinical isolate collection carrying a chromosomal *fosA* gene in the current study. Our collection was notably more phenotypically diverse with ∼70% of isolates being considered a wild-type phenotype, per EUCAST, whereas KPC-producing isolates are generally considered a multidrug-resistant phenotype.

Abbott *et al.* reported the presence of *fosA* in their *K. pneumoniae* isolates (*n* = 20), which was more in agreement with the findings of Elliott *et al.* than the current study.^25^ The IC-production phenotype was unknown for these isolates. Using the disk elution method modified for the current study, 60% (*n*/*N* = 12/20) of Abbott’s isolates screened positive for heteroresistance. The authors were unable to attribute the heteroresistance to an upregulation in *fosA* with quantitative PCR methods. In another study using an *in vitro* bladder infection model, it was shown that baseline heteroresistance among all species *K. pneumoniae* were associated with regrowth and treatment failure. This same treatment failure was not seen among non-heteroresistant *E. coli* despite having an MIC higher than or equivalent to the heteroresistant *K. pneumoniae* that regrew.^24,49^

One of the strengths of the current study is addressing the problem from the three-phenotype approach: NP, IC producers and IC. Previous studies have looked at *fosA* presence in IC producers but failed to address the other two phenotypes to better understand the relationship between IC and *fosA*. When heteroresistance was studied, it was done without the context of IC production. This study is limited by the inclusion of only *K. pneumoniae* and the exclusion of *E. coli*; however, the regrowth in a modelled system (per Abbott *et al.*) with a susceptible MIC phenomenon has not been frequently reported among *E. coli*.^49^ Additionally, due to the volume of IC present among the 116 IC producers we only subcultured and tested the IC closest to the disk, so it is possible that variations in the presence of *fosA* genes among IC may not have been captured. Primer sequences were validated by Liu *et al.* to capture plasmid *fosA3* presence.^13^ Furthermore, the primers we designed were only validated for intrinsic *Klebsiella* spp. and do not assess for variants or other plasmid-mediated *fosA* genes. Future work in assessing the whole genomic sequences of these three isolate types will more accurately assess the presence of and variations within any *fosA* genes. Additionally, studies to determine whether gene expression plays any role in NP isolates screening positive for heteroresistance will be needed.

The production of ≥5 IC among clinical *K. pneumoniae* isolates was common and, in most cases, resulted in a subpopulation with increased MIC. The production of these IC resulted in greater odds of producing a heteroresistant population; however, there was still a notable amount of NP that also had a positive heteroresistance screening. The presence of chromosomal *fosA* appeared to be unrelated to both IC production and a positive heteroresistance screening. The production of IC during fosfomycin DD was a predictor of heteroresistant subpopulations, which is associated with poor outcomes in modelled scenarios. Since fosfomycin susceptibility testing is not endorsed for *K. pneumoniae*, providers should continue to exercise caution to avoid using fosfomycin monotherapy for these infections. Taken together, we conclude that the presence of *K. pneumoniae* IC should prompt avoidance of fosfomycin treatment when IC present during DD testing.
